# The Modulatory Role of Cortisol in the Regulation of Sexual Behavior in Young Males

**DOI:** 10.3389/fnbeh.2020.552567

**Published:** 2020-11-05

**Authors:** Geraldine Rodríguez-Nieto, Alexander T. Sack, Marieke Dewitte, Franziska Emmerling, Teresa Schuhmann

**Affiliations:** ^1^Department of Cognitive Neuroscience, Faculty of Psychology and Neuroscience, Maastricht University, Maastricht, Netherlands; ^2^Department of Movement Sciences, Catholic University of Leuven, Leuven, Belgium; ^3^Department of Clinical Psychology, Faculty of Psychology and Neuroscience, Maastricht University, Maastricht, Netherlands; ^4^Chair of Research and Science Management, Technical University of Munich, Munich, Gemany

**Keywords:** cortisol, sexual regulation, approach-avoidance, medial prefrontal cortex, mood, inhibition, sexual inhibition, sexual arousal

## Abstract

The proneness to be sexually aroused, to perform sexual acts, or to be sexually disinhibited during a particular mood varies across individuals. However, the physiological mechanisms underlying this specific and variable relationship between mood and sex-related processes are poorly understood. We propose that cortisol may act as an important moderator in this as it has shown to influence sexual arousal and to play a neuromodulatory role during emotion regulation. Here, we conducted a functional magnetic resonance imaging study in a sample of young males to investigate whether cortisol modulates the neural response during the approach of sexual stimuli in an approach-avoidance task and whether this potential relationship explains the individual differences in sexual inhibition and in mood-related sexual interest and activity. We revealed that cortisol associates with the anteromedial prefrontal cortex activation during the approach towards sexual stimuli. Moreover, this anteromedial prefrontal cortex response was dependent on individual differences in sexual inhibition and the improvements of negative mood as a result of sexual activity. The anteromedial prefrontal cortex is already known to process bottom-up information, reward, and risk estimation. The neuromodulatory role of cortisol within this region during sexual approach may represent a previously unknown yet key element in the regulation of sexual behavior in young males.

## Introduction

The regulation of sexual desires and behavior is essential to maintain health and social harmony. An impaired ability to regulate sexual behavior can result in a wide spectrum of undesired consequences including sexual risk behavior, hypersexuality, and sexual offense. Dysregulated sexual behavior can be caused by an imbalance between the proneness to be sexually aroused (sexual excitation) and the capability to inhibit such arousal (sexual inhibition; Bancroft and Janssen, 2000), the latter of which has an impact on motivational tendencies, reducing the probability of sexual approach behaviors.

Among the various factors influencing sexual regulation and sexual approach behaviors, the emotional state, i.e., mood, has been suggested to play an important role (Bancroft et al., 2003a,b). However, current evidence reveals conflicting results, pointing to a facilitative, inhibitory, or even no effect of negative mood (e.g., anxiety and stress) on sexual arousal, sexual interest, and sexual activity (Mitchell et al., 1998; Crepaz and Marks, 2001; Koukounas and McCabe, 2001; Nobre et al., 2004; Bradford and Meston, 2006; Bodenmann et al., 2010). This inconsistency may be partly due to individual differences in the proneness to experience sexual arousal when being in a negative mood. In turn, these differences seem to underlie an association with the manifestation and regulation of sexual behavior. Men who experience sexual arousal when being sad or depressed, report a higher number of sexual partners and lifetime one-night-stands (Bancroft et al., 2003a,b). Concerning sexual regulation, sex addicts report to have an increased interest to engage in sexual behavior during states of depression or anxiety (Bancroft and Vukadinovic, 2004) and show higher comorbidity with mood disorders (Reid et al., 2011; Kühn and Gallinat, 2016). Similarly, high levels of hypersexuality symptoms tend to be related to high levels of neuroticism in non-diagnosed men and women (Young, 2008; Rettenberger et al., 2016). Furthermore, male sex offenders are more likely to respond to stress with sexual fantasies or act, putting them at increased risk of sexual recidivism during certain emotional states (McKibben et al., 1994; Hanson and Harris, 2000; Cortoni and Marshall, 2001).

The physiological mechanisms underlying the relationship between sexual approach behaviors and mood may be partly based on the hypothalamic-pituitary-adrenal (HPA) axis. During stressful circumstances, the release of cortisol activates the sympathetic system preparing the body for fight/flight responses. Cortisol also influences cognitive processes such as learning, memory, and emotional arousal processing, which in turn influence approach-avoidance tendencies and behavior (Wolf, 2009; Kaldewaij et al., 2016). Regarding sexual cognition, baseline cortisol levels positively relate to the degree of sexual arousal induced by sexual thoughts (Goldey and van Anders, 2012) and to the amount of physical effort made to visualize female erotic images in men (Chumbley et al., 2014). By increasing the arousal towards emotional salient stimuli, cortisol levels may impact the regulation of sexual arousal and sexual approach behavior. A disruption in the HPA axis potentially leads to a dysregulation of homeostatic processes including sexuality. For instance, hypersexual individuals present a higher rate of irregular HPA endocrine suppression and they have higher levels of the adrenocorticotropic hormone as compared to control individuals. Interestingly, they show an unexpected pattern regarding their cortisol levels, as these are negatively correlated with the severity of their disorder. These patients may engage in more intense and frequent sexual activity to compensate for an irregular endogenous mechanism. Notably, these patients report more depressive symptoms and more childhood trauma (Chatzittofis et al., 2016).

Beyond the changes induced in the autonomic nervous system, the HPA axis activity also yields important effects in the central nervous system. In the brain, limbic structures and the medial prefrontal cortex (mPFC)—essential in processing and regulating emotional arousal (Phelps and LeDoux, 2005; McKlveen et al., 2015)—are rich in glucocorticoid receptors (Sánchez et al., 2000; Ulrich-Lai and Herman, 2009). An increasing number of neuroimaging studies show that cortisol levels modulate the activity and connectivity of these regions during resting state and emotion regulation paradigms (Urry et al., 2006; Kern et al., 2008; Henckens et al., 2012; Veer et al., 2012; Kogler et al., 2016). The mPFC has been assigned a crucial role in the regulation of the HPA axis and has been suggested to coordinate different cognitive functions to create context-specific responses (McKlveen et al., 2015). Its connectivity with the amygdala during approach-avoidance behaviors points to a regulatory feedback mechanism (Volman et al., 2011). The connection between the mPFC and the amygdala also mediates the stress response and shows abnormal patterns in mood disorders such as anxiety disorder and depression (Pagliaccio et al., 2015).

These lines of evidence converge on the notion that cortisol plays a role in neuromodulating brain regions involved in emotion regulation such as the amygdala and mPFC. Moreover, cortisol levels have not only been implicated in the behavioral response towards stress and mood but also in sexual arousal. Therefore, it may be hypothesized that cortisol is involved in the physiological and neural mechanisms underlying the relationship between mood and sexual regulation. In the current study, we first aimed at replicating the relationship between cortisol and sexual arousal and to examine whether this relationship could explain a potential co-occurrence of sexual arousal with emotional states that elicit a fight-flight response (anxiety, stress, and anger). Importantly, we also aimed to investigate if cortisol directly modulates the neural activity during sexually motivated approach behavior. For these purposes, we implemented an Approach-Avoidance paradigm to simulate natural motivational tendencies towards affective stimuli. As the neuromodulatory role of cortisol in sexual cognition has not been studied yet, we performed a whole-brain analysis, although we were particularly interested in the modulating role of cortisol within the mPFC and its interaction with the amygdala. Finally, we also investigated whether potential brain activity modulated by cortisol during sexual approach-avoidance behaviors relates to sexual regulation traits. More specifically, we examined sexual inhibition traits and the degree to which individuals’ sexual interest and behavior are associated with mood (i.e., anger, sadness, and anxiety). To assess sexual inhibition we considered the second factor of sexual inhibition from the dual control model of sexual response. This factor refers to the proneness of inhibiting a sexual response due to possible negative consequences (e.g., contagion of sexual transmission disease; Bancroft and Janssen, 2000) and has therefore been shown to be related to risky and out-of-control sexual behaviors (Bancroft and Vukadinovic, 2004; Bancroft et al., 2004). To assess individual differences in the relationship between mood-sexual interest and behavior, we considered the effect of mood on sexual desire, the benefits on mood from sexual activity, and the effects of mood on regrettable sexual behavior (Janssen et al., 2013). Finally, in this study, only men were included because women and men respond differently concerning sexual cognition (Dewitte, 2016; Sjoberg and Cole, 2018) and because women are less prone to sexual inhibition impairments such as hypersexuality and sexual offense (Kuzma and Black, 2008; Knack et al., 2015). Similarly, because our approach included visual heterosexual erotic stimuli, we only included heterosexual participants.

## Materials and Methods

The current analyses are derived from a dataset that was used to compare the neural mechanisms of different inhibitory processes (see Rodríguez-Nieto et al., 2019a). The research question and goal of the current study are not related to the previous one and therefore all the here analyses presented here and associated results have not been reported elsewhere.

### Participants

Twenty-five healthy male right-handed participants without neurological or psychiatric disorders participated in this study. Participants were screened for disorders and medication intake and none reported having a disease or taking medication that could alter cortisol levels. Likewise, participants did not have excessive over or underweight, which could indicate endocrine dysregulation. Twenty-four participants declared a heterosexual orientation and one participant chose not to give information regarding his sexual orientation. One participant was excluded due to excessive head motion, and a second participant due to technical difficulties leading to an incomplete data set (final sample: *N* = 23, mean age = 24.77, SD = 4.76, range = 18–35 years). Participants gave written informed consent and were financially reimbursed. This study was approved by the Ethical Committee of the Faculty of Psychology and Neuroscience at Maastricht University (Approval no. ECP 142-01-07-2014).

### Procedure and Instruments

Participants were asked to abstain from eating or drinking (anything besides water), brushing their teeth, and vigorously exercising 45 min before their appointment and were recommended to drink water 10 min before to facilitate saliva collection. The experiment took place between 8:30 and 10:30 in the morning. Upon the participant’s arrival and after signing the informed consent, participants were instructed to drool their saliva into a 3.6 ml hormonal tube assay with the aid of a small straw.

Following the saliva collection and before entering the scanning room, participants filled in a short scale regarding their mood states and performed an Approach-Avoidance practice task with non-sexual stimuli (animals and plants) to reassure their understanding of the instructions. After the collection of functional and anatomical MRI data, participants were asked to fill in the self-reports on the computer. Participants were identified with a four digits number as their ID to increase their confidence over the anonymity of their answers. After the experiment, saliva samples were stored at 4°C.

### Questionnaires

#### Revised Mood and Sexuality Questionnaire (MSQ-R; Janssen et al., 2013)

This instrument assesses the likeliness of experiencing sexual interest and arousal, masturbating, or performing a sexual act that could later be regretted, when being in a particular mood (anxiety, sadness, anger, or happiness). Also, it assesses the effects of sexual activity on mood and attachment. Participants answered on a five-point Likert scale ranging from *Much less than usual* to *Much more than usual* (e.g., “When I feel angry I think about sex…”). For this study, we considered the following indices: (a) Effects of anxiety on sexual desire—Refers to the effects of anxiety over sexual desire and interest (*AnxDes; Possible range: 2–10*); (b) Positive effects of sex on mood—Refers to the benefits on mood from sexual activity (i.e., decreasing negative mood, increasing attachment with a partner, and feeling better about oneself; *PositFx; Possible range: 9–45*); and (c) Effect of negative mood on regrettable sexual behavior—Concerns the effects of negative mood (anger, sadness, and anxiety) on the likelihood of doing something sexual that might be regretted later (*RegrSx; Possible range: 3–15*).

#### Sexual Inhibition/Sexual Excitation Scales (SIS/SES; Janssen et al., 2002)

This 45 items-scale measures the individual propensity for sexual inhibition and excitation. We only considered the second factor of sexual inhibition (SIS2) which assesses inhibition (unlikeliness to stay aroused or to maintain an erection) due to the threat of performance consequences (e.g., risk of being caught, unwanted pregnancy, sexually transmitted diseases). Answers were registered on a four-point Likert scale (ranging from 1 = *strongly agree* to 4 = *strongly disagree; Possible range: 11–44, with a higher score indicating higher inhibition*).

#### Mood and Task Effects

Before entering the scanner participants answered to what extent they felt in a particular mood (happy, anxious, sad, stressed, relaxed, excited, angry) on a five-point Likert scale (*Not at all—Very much*). After the scanning, they filled in a SAM (self- assessment manikin; Bradley and Lang, 1994) type questionnaire indicating to what extent they liked or disliked the task (valence), to what extent they felt aroused as a result of the task execution (arousal) and to what extent they felt in control over the stimuli during the task (dominance). To answer, they could select one of five images which ranked from *Very pleasant to Very unpleasant* (valence), *Very arousing to Not arousing* (arousal), and *High control to Low control* (dominance).

### Cortisol Assay

After shipping, cortisol samples were frozen at −20°C. Analyses were performed at the University Hospital of Ghent, Belgium. Cortisol concentrations in saliva (in μg/dl) were obtained by high-performance liquid chromatography-tandem mass spectrometry (LC-MS/MS). An AB Sciex 5,500 triple-quadrupole mass spectrometer (AB Sciex; Toronto, Canada) was coupled with an Atmospheric Pressure Chemical Ionization (APCI) probe on the Turbo-V source. Intra-assay coefficient of variation was 4.6% at 0.21 μg/dl. The lower limit of quantification (LOQ) of this method was 0.01 μg/dl.

### Approach-Avoidance Task

This paradigm assesses approach-avoidance tendencies towards emotional stimuli. Previous versions with sexual content proved sensitive to gender differences (Dewitte, 2016), to the viewing time of other erotic stimuli (Hofmann et al., 2009), and to predict the frequency of pornography watching (Rodríguez-Nieto et al., 2019b).

**Figure 1:**
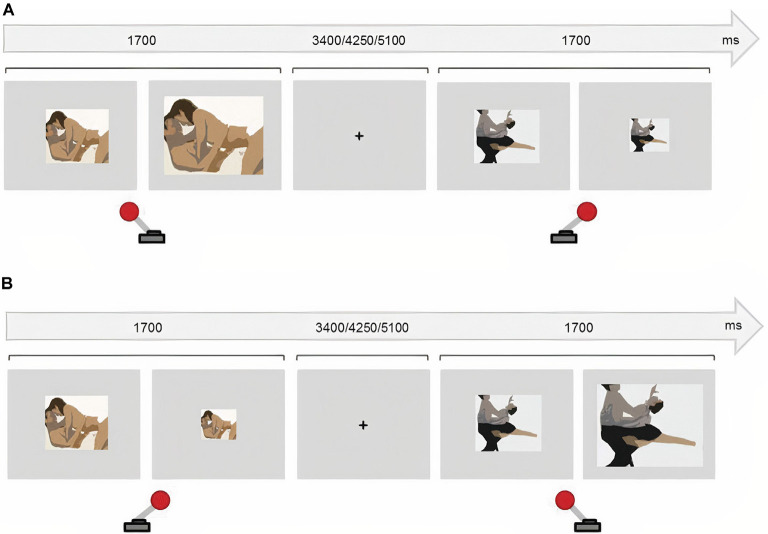
Approach-Avoidance task. **(A)** In congruent blocks, participants approached sexual stimuli (pulling the joystick towards them) and avoided (push the joystick away) non-sexual stimuli. **(B)** In incongruent blocks participants avoided sexual stimuli and approached non-sexual stimuli.

The current version consisted of four counterbalanced blocks of 48 randomized trials each. In half of the blocks, participants were instructed to approach the sexual stimuli (50% of the trials) and to avoid the non-sexual stimuli. Participants were instructed to do the opposite in the other two blocks. To approach the stimuli, participants pulled a joystick towards them which doubled the picture size. Participants avoided the stimuli by pushing the joystick away, which halved the picture size. The images were always resized based on participants’ joystick movements regardless of the instruction. Incorrect trials (e.g., approaching a sexual image during a Sex Avoid run) and omission errors were discarded from the data. The presentation of every stimulus lasted 1,700 ms and the resizing occurred 1,300 ms after the presentation onset, to avoid variability across trials and participants. An inter-trial fixation cross was presented for 3,400, 4,250, or 5,100 ms (Figure 1).

As sexual stimuli, color photographs of heterosexual couples having intercourse or oral sex were used. Ninety-five percentage of these stimuli were previously evaluated in terms of valence and arousability (Rupp and Wallen, 2007). The additional pictures were selected from the internet and three judges evaluated them to ensure similar features to the previously rated images (regarding body postures and dimensions). Non-sexual stimuli were color photographs of a woman and a man dancing (Dewitte, 2016). The proportion of body exposition (with special attention to the female body) concerning the whole picture was comparable in both conditions. Due to the limited number of evaluated images, in half of the blocks the default size of the pictures was 337.9 × 272.5 pixels (horizontal orientation) and in the other half was 257.6 × 400 pixels (vertical orientation). Images were displayed on a light gray background. The task was programmed in PsychoPy (Peirce, 2007).

### Behavioral Data Analysis

According to our research questions, the following were our variables of interest: Sexual Inhibition factor 2 (SIS2), effects of anxiety in sexual desire (MSQ-R Anx Des), positive effects of sex on mood (MSQ-R PositFx), effects of negative mood on regrettable sexual behavior (MSQ-R RegrSx), mood prior entering the scanner (Happy, Anxious, Sad, Stressed, Relaxed, Excited and Angry), Valence and Dominance regarding the stimuli from the sexual task and Arousal evoked from the sexual task.

We report descriptive statistics for the cortisol measurements and our variables of interest. Before correlating cortisol with our variables of interests, we relied on normality Shapiro-Wilk tests and visual inspection of the data distribution to know which correlations had to be parametric (Pearson) vs. non-parametric (Spearman). According to our hypotheses, we also correlated Arousal and Mood (Anxious, Stress, Angry), and calculated partial correlations to control for the effect of cortisol as confound. The behavioral statistical analyses were executed with R (version 3.5.1) software (R Core Team, 2016).

### fMRI Acquisition and Pre-processing

Data were acquired at the 3 T Siemens Prisma Scanner at the Maastricht Brain Imaging Center, Maastricht University. High-resolution anatomical images were acquired with an MPRAGE sequence (TR = 2,250 ms, TE = 2.21 ms, FOV = 256 mm, 192 sagittal slices, isovoxel 1 mm^3^). Functional EPI images were collected using an in-house developed multi-echo multi-band sequence (TR = 850 ms, TE = 15/30/44 ms, flip angle = 50°, FOV = 210 mm, 36 slices, isovoxel 3 mm^3^). Each stimulus lasted for 1,700 ms and its onset coincided with the start of the first repetition time-period. The image resizing was locked at 1,300 ms, regardless of the reaction times of participants, to control for visual input between trials and participants. Online scanner reconstruction was performed using the slice-GRAPPA algorithm (Setsompop et al., 2012) with leakage artifact reduction (Cauley et al., 2014) as implemented in the reconstruction of the MGH blipped-CAIPI SMS-EPI distribution (software and complete documentation are available^1^). This GRAPPA sequence was used to optimize the BOLD signal in frontoventral regions.

The imaging data were pre-processed and analyzed with Brain Voyager QX Version 2.8.4.2645 (Brain Innovation, Maastricht, Netherlands).

Before the pre-processing, the echo images were combined using an optimized echo weighting method (Poser et al., 2006). The images were motion-corrected (trilinear/sinc interpolation and aligned to the first functional volume acquired after the anatomical sequence) and corrected for slice timing skew using temporal sinc interpolation. A temporal high pass filter (three cycles) was applied. Images were co-registered to the individual T1 weighted images and normalized to Talairach stereotaxic space. Volume time courses were spatially smoothed using a 6 mm full width half maximum Gaussian kernel.

### fMRI Data Analyses

To analyze the activation pattern of every task an event-related approach was implemented using a GLM model and a random-effects group analysis. Motion correction parameters were included as confound variables. Whole-brain contrast analysis was conducted to compare the sexual approaching trials with the sexual avoiding trials including the cortisol levels as a covariate. The resulting activations were corrected for multiple comparisons using cluster threshold level estimation (1,000 Monte Carlo simulation iterations; Forman et al., 1995). The nomenclature of the cluster peak values was defined with the software tool Talairach Client (Lancaster et al., 1997, 2000). We also conducted a GLM analysis with the same characteristics but adding the endogenous cortisol levels as a covariate; the resulting ANCOVA map was corrected for multiple comparisons with 1,000 Monte Carlo iterations. We generated a mask from the resulting activation in the amPFC, from which we extracted the individual model parameters (beta) from the same model (Sex Approach > Sex Avoid, covariate: cortisol). We performed correlation analyses of the beta parameter with the SIS2 and MSQ-R variables. To examine the specificity of our results, we also correlated the beta parameters with sexual excitation (SES).

### Functional Connectivity Data Analysis

To investigate the functional connectivity of the amPFC during the approach of sexual stimuli, we used the mask generated in the previous analysis as a seed in psychophysiological interaction (PPI) analysis (Friston et al., 1997). As we were particularly interested in the functional connectivity with the amygdalae, we used an anatomically defined bilateral mask for this region (from Brain Voyager QX Version 2.8.4.2645). The resulting activations were corrected for multiple comparisons using cluster threshold level estimation (1,000 Monte Carlo simulation iterations; Forman et al., 1995).

## Results

### Cortisol, Mood, and Traits

Participants cortisol levels mean was 0.31 (SD = 0.21) μg/dl (or 8.55 nmol/l, SD = 5.79) which is within the morning reference range for this sample (range: 0.11- 0.74 μg/dl or 3.1–20.5 nmol/l; Aardal and Holm, 1995). Participants’ average score in the SIS2 was 25.17 (SD = 6.58). MSQR scores were as follows: Mean Anxiety-Sexual Desire: 4.61 (SD = 1.83), Mean Sadness-Sexual Desire: 4.84 (SD = 2.11), Mean Anger-Sexual Desire: 4.91 (SD = 2.13), with a higher score indicating a stronger relationship. Concerning positive effects derived from sexual activity while being angry, sad, or anxious, the average score was 7.08 (SD = 3.37). On average participants reported mild arousal as a result of the execution of the task (mean score = 3.35, SD = 1.07). These results are summarized in Table 1. The correlations between cortisol and self-report scores are shown in Table 2. According to what we expected, participants with higher cortisol levels reported higher arousal in response to the task. Although cortisol did not show a significant correlation with the degree of anxiety or stress reported before entering the scanner, it showed a trend towards significance with anger. Also, cortisol levels correlated positively with the levels of sadness and negatively with happiness and excitation reported before entering the scanner. Contrary to our expectations, the degree of arousal reported after the sexual task did not relate to anger, stress, or anxiety (Table 2). On the contrary, arousal correlated with sadness (ρ = 0.51, *p* = 0.02), but this correlation was no longer significant after controlling for cortisol levels (ρ = 0.34, *p* = 0.11).

**Table 1:** Average of cortisol levels and self-reports.

	Cortisol	SIS2	MSQ-R AnxDes	MSQ-R PositFx	MSQ-R RegrSx	Valence	Arousal	Dominance
Mean (SD)	0.31 (0.21)	25.17 (6.58)	4.61 (1.83)	23.34 (9.19)	1.82 (1.03)	2.56 (0.72)	3.35 (1.07)	2.35 (0.88)
	**Happy**	**Anxious**	**Sad**	**Stressed**	**Relaxed**	**Excited**	**Angry**
Mean (SD)	3.22 (1.11)	1.59 (0.96)	1.22 (0.68)	1.72 (0.82)	3.41 (0.85)	3 (0.98)	1.13 (0.46)	

*SIS2, Sexual Inhibition (second factor); MSQ–R, Revised Mood and Sexuality Questionnaire; AnxDes, Degree of sexual interest experienced while being anxious; PositFx, Positive effects of sexual activity over mood; RegrS, Likelihood of regrettable sexual behavior while being in a negative mood state*.

**Table 2:** Correlations between cortisol levels and self-reports.

	SIS2	MSQ-R AnxDes	MSQ-R PositFx	MSQ-R RegrSx	Valence	Arousal	Dominance
Cortisol (*p*)	−0.02^p^ (0.91)	−0.19^p^ (0.37)	−0.11^p^ (0.61)	0.03^s^ (0.87)	0.17^s^ (0.43)	0.44^s^ (0.03)	0.27^s^ (0.19)
	**Happy**	**Anxious**	**Sad**	**Stressed**	**Relaxed**	**Excited**	**Angry**
Cortisol (*p*)	−0.61^s^ (0.002)	−0.13^s^ (0.55)	0.55^s^ (0.007)	−0.07^s^ (0.73)	−0.36^s^ (0.09)	−0.77^s^ (0.001)	0.38^s^ (0.08)

*SIS2, Sexual Inhibition (second factor); MSQ–R, Revised Mood and Sexuality Questionnaire; AnxDes, Degree of sexual interest experienced while being anxious; PositFx, Positive effects of sexual activity over mood; RegrSx, Likelihood of regrettable sexual behavior while being in a negative mood state. Valence, Arousal, Dominance, Rating given by the subjects to the performance of the task. Happy, Anxious, Sad, Stress, Relaxed, Excited, Angry, Degree to which participants experienced being in that mood before the scanning session. p, Pearson correlation. s, Spearman correlation*.

### Brain Correlates of Sexual Approach With Cortisol

**Figure 2:**
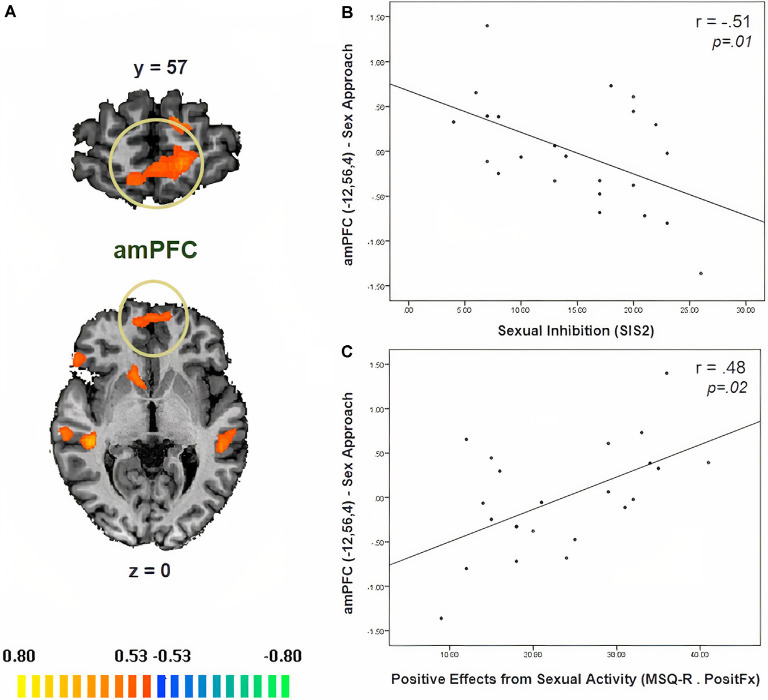
**(A)** Neural correlates of cortisol during the approach of sexual stimuli as compared to the avoidance of the same stimuli (*p* = 0.01, CLTC). **(B)** The activity of the anteromedial prefrontal cortex (amPFC) during the approach of sexual stimuli negatively correlated with sexual inhibition (higher SIS2 scores indicate higher sexual inhibition). **(C)** The amPFC activity during the Sex Approach condition also related (positively) with the degree to which participants reported mood improvement (when being sad, anxious, or angry) as a result of sexual activity (*MSQ R—PositFx)*. Activation is displayed on the anatomical data of one participant from the sample.

In Table 3, we display the regions that were active during the sexual approach as compared to sexual avoidance. These regions include the amygdala, the hippocampus, the parahippocampal gyri, and visual processing regions [*p* = 0.005, CLTC (cluster-level threshold correction)]. Endogenous cortisol levels positively correlated with activation in the anteromedial prefrontal cortex (amPFC), the insula, the superior temporal gyrus, the precentral gyrus, and the precuneus when participants approached sexual stimuli, as compared to the avoidance of the same stimuli (*p* = 0.005, CLTC; Table 4; Figure 2A). At a more liberal threshold, cortisol levels positively correlated with the activity in the bilateral inferior and superior frontal gyri, the middle temporal gyrus, the inferior occipital gyrus, and the caudate (*p* = 0.01, CLTC; Table 4). The model parameters (beta) from the area of the amPFC modulated by cortisol during the approach of sexual stimuli were extracted for every participant. These parameters were negatively correlated with sexual inhibition (SIS2; *r* = −0.51, *p* = 0.01; Figure 2B) and positively with the degree to which participants reported mood improvements (when being sad, anxious, or angry) as result of sexual activity (*MSQ R—PositFx*; *r* = 0.48, *p* = 0.02; Figure 2C). To address specificity, we also conducted a correlation analysis between sexual excitation (SES) scores and the amPFC activation parameters, which was not significant (*r* = 0.31, *p* = 0.14).

**Table 3:** Active regions during the approach of sexual stimuli as compared to the avoidance condition.

	Sex approach > sex avoid						
	BA	*x*	*y*	*z*	Size (mm^3^)	*t*	*p*
Lingual gyrus	18	−9	−76	−2	66,855	9.92	0.00001
Parahippocampal gyrus	28	27	−19	−8	360	4.58	0.0001
Amygdala		21	−7	−8	917	5.01	0.00005
Parahippocampal gyrus	30	18	−40	−5	397	4.22	0.0003
Hippocampus		−27	−13	−11	3,776	5.68	0.00001
Middle occipital gyrus	19	−45	−82	4	619	3.81	0.0009

*CLTC 0.005*.

**Table 4:** Brain activations correlating with endogenous cortisol during the approach of sexual stimuli.

	Sex approach > sex avoid						
	BA	*x*	*y*	*z*	Size (mm^3^)	*r*	*p*
Sex approach-sex avoid							
Insula	22	42	−28	−2	1,514**	0.73	0.00005
Superior temporal gyrus	22	51	−13	−8	891**	0.76	0.00002
Precuneus	19	42	−70	40	1,443**	0.71	0.0001
Precentral gyrus	9	36	8	37	705**	0.69	0.0002
Medial frontal gyrus	10	−12	56	4	782**	0.65	0.0006
Posterior Lobe/Cerebellum		−27	−79	−27	1,107**	0.68	0.0002
Superior temporal gyrus	22	−48	−34	1	715**	0.65	0.0007
Inferior frontal gyrus	45	51	26	7	1,386*	0.63	0.001
Middle temporal gyrus	19	36	−64	19	771*	0.65	0.0006
Inferior occipital gyrus	17	18	−91	−8	1,104*	0.73	0.00007
Superior frontal gyrus	9	21	35	34	1,097*	0.68	0.0003
Caudate (Head)		9	14	−2	763*	0.65	0.0007
Inferior frontal gyrus	46	−45	29	7	827*	0.69	0.0002

**CLTC *p* < 0.001; *CLTC *p* < 0.01.

### Functional Connectivity

**Figure 3:**
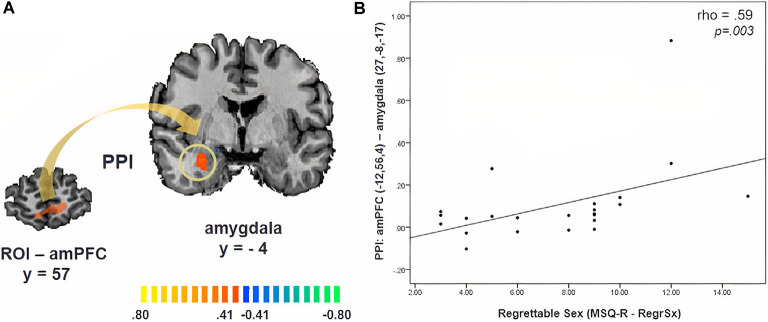
**(A)** Psychophysiological interaction (PPI) map revealing functional connectivity between the anteromedial prefrontal cortex (amPFC) and the amygdala during the approach of sexual stimuli. **(B)** The strength of this functional connectivity is related to the degree to which participants reported being likely to commit a potentially regrettable sexual act while being in a negative mood (angry, sad, or anxious; MSQ R—RegrSx). Activation is displayed on the anatomical data of one participant from the sample.

PPI analysis revealed significant functional connectivity between the amPFC (*x, y, z* = −12, 56, 4, ROI size: 782 mm^3^) and the right amygdala during the approach of sexual stimuli in comparison to its avoidance, which was not modulated by cortisol (*x, y, z* = 27, −8, −17, cluster size: 504 mm^3^, *p* = 0.05, CLTC; Figure 3A). Participants with a stronger amPFC-amygdala coupling reported an increased likelihood of performing a regrettable sexual act while being in a negative mood (angry, sad, or anxious; *MSQ R- RegrSx*; ρ = 0.59, *p* = 0.003; Figure 3B).

## Discussion

In this study, we investigated the role of cortisol regarding the relationship between mood and arousal in a sexual context as well as the neuromodulatory role of cortisol during sexual approach behavior in young males. Although we replicated the relationship between cortisol and the arousal induced by sexual cognition, we did not find evidence for the hypothesis that this association underlies the relationship between sexual arousal and negative mood states. Regarding the neuromodulatory role of cortisol, we observed that higher levels of cortisol were related to increased activation in the amPFC, the insula, the superior temporal gyrus, the precentral gyrus, and the precuneus during sexual approach behavior. During this condition, the amPFC was functionally connected to the right amygdala. Remarkably, the activation in the amPFC was negatively related to sexual inhibition traits and positively related to the benefits of mood from sexual activity. Also, the strength of the connectivity between the amPFC and the amygdala is related to the extent to which individuals reported the likeliness of doing a regrettable sexual act while being in a negative mood.

### Relationship Between Cortisol, Sexual Arousal, and Mood

Our results showed a positive relationship between endogenous baseline cortisol levels and the reported level of arousal evoked by the sexual task, which is consistent with previous evidence showing a positive relationship between cortisol and sexual arousal (Goldey and van Anders, 2012). This association can be explained from a functional perspective because cortisol activates the sympathetic autonomic system and promotes fast metabolization to generate energy which is needed to facilitate sexual arousal. We aimed to examine whether the relationship between cortisol and arousal in a sexual context, could also underlie the relationship between arousal and mood states that elicit a flight/fight response, as both arousals in a sexual context and states of anxiety/stress/anger are characterized by a high heart rate and blood pressure (Goldey and van Anders, 2012). We did not find a relationship between most mood states and the arousal evoked by sexual cognition. Only sadness was directly related to arousal, but this relationship was no longer significant when controlling for cortisol. Also, we did not find a relationship between cortisol levels and the levels of anxiety, anger, or stress reported by participants before entering the scanner. These results showed that on average, negative moods are not enough to elicit enhanced arousal in a sexual context. Remarkably, high baseline cortisol levels seem sufficient to facilitate this sort of arousal. We did not measure blood pressure and heart rate, so we cannot conclude that the relationship between cortisol and sexual desire is associated with the manifestation of autonomic responses. Based on our data, we also cannot discard a possible association between negative mood states and sexual desire mediated by autonomic responses. The lack of association between negative mood states and arousal in a sexual context can also be attributed to individual differences, as some individuals are more prone to experience sexual arousal when being angry, sad, or anxious (Bancroft and Vukadinovic, 2004).

### Cortisol, Brain Response, and Psychological Traits

In the sexual approach condition, individuals higher in cortisol showed an increased brain response in the precentral gyrus, the caudate, and the insula. This may indicate a stronger proneness to act (approach the sexual stimuli) accompanied by an enhanced visceral sensitivity. Likewise, increased activity in the bilateral inferior frontal gyrus was associated with higher cortisol levels. The inferior frontal gyrus is engaged in the detection of salient stimuli and its temporal disruption leads to an increase in inhibition of sexual cues (Erika-Florence et al., 2014; Rodriguez et al., 2018). Thus, *via* this circuit, cortisol may increase the perception sensitivity towards arousing relevant cues in the environment. In the same condition (approaching sexual stimuli) cortisol is related to an increased response in supramodal regions: the amPFC, precuneus, and the superior temporal gyrus. The amPFC cortisol-modulated pattern during the sexual approach condition as compared to sexual avoidance implies a negative modulation during the sexual avoidance condition (i.e., less amPFC activity according to more cortisol levels during sexual avoidance as compared to sexual approach) in which individuals restrain a motivationally-driven motor response. This is in concordance with previous evidence where higher levels or non-normative circadian patterns of cortisol related to a decrease of amPFC activity during emotion regulation compared to an emotional but non-regulatory condition (Urry et al., 2006; Kern et al., 2008). Thus, the higher activation of the amPFC during the non-regulatory condition associated with higher cortisol levels may indicate a higher demand to control autonomic responses. In line with this, individuals higher in cortisol reported being more sexually aroused by the task and they also showed an increased amPFC response while approaching sexual stimuli.

Remarkably, individuals with a higher neural response of the amPFC during the approach sexual condition reported being more sexually disinhibited and reported benefits on mood (improvement in the sad, angry, or anxious mood) as a consequence of the sexual activity. Whereas the mPFC seems to play a role in the regulation of the HPA axis, its most anterior portion (i.e., frontal pole) is associated with higher-order processing such as the maintenance of higher-order goals, hierarchical processing, the anticipation of consequences, mentalizing, and self-reflection (Ramnani and Owen, 2004; Burgess et al., 2007; Roca et al., 2011). These functions are not mutually exclusive, as the hierarchical processing and goal maintenance can switch according to environmental demands which in turn require the organism to adapt (including autonomic responses). Accordingly, it has also been argued that the vmPFC (overlapping with our amPFC cluster) integrates high social values with basic emotional processes (e.g., visceroaffective), thereby modulating approach-avoidance behaviors (Bzdok et al., 2013). Our results show that individuals who are less likely to inhibit their sexual arousal neglecting higher order values and/or long term goals (e.g., having sex despite pain or health risks) show an increase in amPFC when they approached sexual stimuli. This counterintuitive finding (i.e., enhanced regulation activity in more disinhibited individuals) may reflect a higher demand to control autonomic responses during a sexually arousing condition, in particular in a context where individuals cannot unfold their sexual response. Alternatively, the activation of this region, by processing bottom-up information and memory-informed reward and risk estimation (Bzdok et al., 2013) may be reflecting a stronger weighting of rewarding stimuli, reducing risk perception, and favoring approach behaviors.

The amPFC also is shown to be more active in individuals who reported higher benefits on mood (e.g., feeling less angry, sad, or anxious, or feeling better about themselves) from sexual activity. It has been suggested that some individuals use sex to cope with their mood, as sexual activity can act as an anxiolytic (Bancroft and Vukadinovic, 2004). An enhanced amPFC activation could be reflecting that individuals who reported higher benefits on mood from sex recruit more bottom-up regulatory mechanisms (immediately rewarding and potentially anxiolytic) to cope with negative emotional states, perhaps as opposed to recruiting more dorsal top-down processes.

Notably, the amPFC activity was related to sexual inhibition traits but not to sexual excitation. Likewise, the amPFC did not correlate with the degree to which individuals show sexual desire or sexual interest during negative moods, but specifically to the mood ameliorating effects of sexual activity (e.g., feeling less anxious after sexual activity). This observation seems to support the involvement of amPFC in the integration of visceroaffective information in homeostatic mechanisms, and not solely in evaluating or processing the arousal state inputs.

Furthermore, the strength of the functional connectivity between the amPFC and the amygdala while approaching sexual stimuli was associated with the likelihood that participants reported engaging in sexual activity while being in a negative mood that might be subsequently regretted. Once more, it remains unclear whether this neural pattern constitutes an enhanced compensatory regulation mechanism, or an integrative process favoring immediate gratification despite consequences. In the first case, participants who are more likely to perform potentially regrettable acts when being in a negative mood would recruit this circuitry more because their regulatory demands are higher. In the second possibility, enhanced signaling from the amygdala could represent a stronger integration of arousing information, leading to an emotionally driven behavioral output. A previous approach-avoidance study showed a negative coupling between the anterior PFC (a more laterally located cluster relative to ours) and the amygdala while participants overrode a natural avoidance tendency (i.e., approach angry faces), suggesting a downregulation mechanism (Volman et al., 2016). In this study instead, we observed a positive coupling during the non-regulatory condition which may underlie a positive feedback mechanism rather than a downregulation one.

Whereas cortisol did not hold a direct relationship with the degree to which individuals felt in control over the sexual stimuli, with sexual inhibition trait, or mood associated sexual behavior, cortisol levels were associated with the activity in the amPFC during sexual approach behavior. Thus, the amPFC neural response of individuals with higher levels of cortisol resembled the neural activity of sexually disinhibited individuals and individuals who reported an improvement in sadness, anger, or anxiety mood as a consequence of the sexual activity. This seems to indicate that the enhancement of sympathetic reactivity produced by cortisol leads to a higher sexual arousing state that can interfere with the regulation of sexual approach behavior similar to that of individuals with regulatory impairments.

This study is not without limitations. The first limitation of this study is that we only tested young heterosexual males. We decided to study this sample because the proportion of women displaying sexual regulation impairments is significantly less (Kuzma and Black, 2008; Knack et al., 2015). Furthermore, although the literature on the relationship between cortisol, mood, and sexual cognition is scarce, women and homosexuals are even less studied. There are important differences among women, men, and individuals with different sexual orientations concerning sexual cognition and behavior (Bancroft et al., 2003a; Dewitte, 2016). For instance, regarding the relationship between mood and sexuality, heterosexual women are less prone than men (homo and heterosexuals) to experience enhanced sexual arousal when feeling anxious or stressed, and heterosexual women and homosexual men are less likely to experience an increase in sexual desire while feeling sad (Janssen et al., 2013). Therefore, it is not possible to generalize the present findings, and the mechanisms here described remain to be investigated in women and homosexuals.

A second limitation of our study is the relatively small sample size. The main risk of small sample sizes is the commission of false negatives, that is, erroneously reject the null hypothesis. This study showed to have sufficient statistical power to detect our effect of interest (the effect of cortisol in the mPFC activation during sexual approach). Still, it is important to mention that a lack of power also risks the commission of Type II errors, and therefore, our results should be interpreted with caution.

A final limitation is its correlational nature which prevents us to conclude that high cortisol levels increase the regulatory circuitry demands by increasing arousal. Alternatively, high cortisol levels could directly cause an increase in such circuitry as an adaptive response in challenging situations. Furthermore, it remains unclear whether this mechanism favors an approach to sexual behavior by enhanced bottom-up processing of what is momentarily relevant (Bzdok et al., 2013). In this sense, cortisol may enhance the weighting and integration of rewarding and potentially anxiolytic stimuli as a compensatory mechanism to achieve homeostasis. Whereas moderate levels of cortisol are adaptive and even have anxiolytic effects (Soravia et al., 2006; de Quervain and Margraf, 2008), the continuous exertion of the system may lead to abnormal regulatory mechanisms. To this regard, individuals with an irregular HPA axis activity may lack adequate endogenous means and make use of external factors (e.g., sexual activity or substance abuse) to cope with negative mood which eventually could develop into an addiction, as seems to be the case of hypersexual individuals (Chatzittofis et al., 2016).

Future studies may test the different posed hypotheses and examine to what extent they apply in clinical conditions where HPA mechanisms are disrupted. Another interesting avenue for research aiming to understand the relationship between mood and sexual regulation is exploring the interaction of cortisol with monoamines, as serotonin and dopamine have shown to influence both mood and sexual regulation (Kafka, 2010). Likewise, the interaction between cortisol and testosterone has shown to be important in aggression, dominance, and economic decisions (Mehta and Prasad, 2015), but has been scarcely studied in the sexual context. Finally, the Approach-Avoidance paradigm can be tested as a training tool for individuals with sexual regulation impairments. To this respect, emotional training with an Approach-Avoidance paradigm has shown to be successful in socially anxious individuals (Rinck et al., 2013).

## Conclusion

In sum, this study showed that cortisol levels are associated with the response of the amPFC during the sexual approach. Interestingly, the activation in the amPFC and the strength of its connectivity with the amygdala were related to individual differences in sexual inhibition and perceived benefits on mood from sexual activity. These results imply that during the sexual approach, cortisol levels are associated with the activity of brain areas involved in sexual regulation. These circuits may, at least in part, underlie the association between negative emotional states and the dysregulation of sexual behavior –neglecting social values and/or long-term consequences- during arousing situations.

## Data Availability Statement

The raw data supporting the conclusions of this article will be made available by the authors, without undue reservation.

## Ethics Statement

The studies involving human participants were reviewed and approved by the Ethical Committee of the Faculty of Psychology and Neuroscience at Maastricht University. The patients/participants provided their written informed consent to participate in this study.

## Author Contributions

GR-N, AS, MD, FE, and TS made substantial contributions in the design of the study, the data interpretation, and the manuscript revision. GR-N performed the experiments, data analyses, and wrote the manuscript. All authors contributed to the article and approved the submitted version.

## Conflict of Interest

The authors declare that the research was conducted in the absence of any commercial or financial relationships that could be construed as a potential conflict of interest.

## Footnotes

^1^https://www.nmr.mgh.harvard.edu/software/c2p/sms
